# Changes in the Plant β-Sitosterol/Stigmasterol Ratio Caused by the Plant Parasitic Nematode *Meloidogyne incognita*

**DOI:** 10.3390/plants10020292

**Published:** 2021-02-04

**Authors:** Alessandro Cabianca, Laurin Müller, Katharina Pawlowski, Paul Dahlin

**Affiliations:** 1 Agroscope, Research Division, Plant Protection, Phytopathology and Zoology in Fruit and Vegetable Production, 8820 Wädenswil, Switzerland; alessandro.cabianca@agroscope.admin.ch (A.C.); laurin.mueller@agroscope.admin.ch (L.M.); 2 Department of Ecology, Environment and Plant Sciences, Stockholm University, 106 91 Stockholm, Sweden; katharina.pawlowski@su.se

**Keywords:** sterol, β-sitosterol, stigmasterol, plant parasitic nematode, *CYP710A*, 22C-desaturase

## Abstract

Sterols play a key role in various physiological processes of plants. Commonly, stigmasterol, β-sitosterol and campesterol represent the main plant sterols, and cholesterol is often reported as a trace sterol. Changes in plant sterols, especially in β-sitosterol/stigmasterol levels, can be induced by different biotic and abiotic factors. Plant parasitic nematodes, such as the root-knot nematode *Meloidogyne incognita,* are devastating pathogens known to circumvent plant defense mechanisms. In this study, we investigated the changes in sterols of agricultural important crops, *Brassica juncea* (brown mustard), *Cucumis sativus* (cucumber), *Glycine max* (soybean), *Solanum lycopersicum* (tomato) and *Zea mays* (corn), 21 days post inoculation (dpi) with *M. incognita*. The main changes affected the β-sitosterol/stigmasterol ratio, with an increase of β-sitosterol and a decrease of stigmasterol in *S. lycopersicum*, *G. max*, *C. sativus* and *Z. mays*. Furthermore, cholesterol levels increased in tomato, cucumber and corn, while cholesterol levels often were below the detection limit in the respective uninfected plants. To better understand the changes in the β-sitosterol/stigmasterol ratio, gene expression analysis was conducted in tomato cv. Moneymaker for the sterol 22C-desaturase gene *CYP710A11*, responsible for the conversion of β-sitosterol to stigmasterol. Our results showed that the expression of *CYP710A11* was in line with the sterol profile of tomato after *M. incognita* infection. Since sterols play a key role in plant-pathogen interactions, this finding opens novel insights in plant nematode interactions.

## 1. Introduction

**Figure 1:**
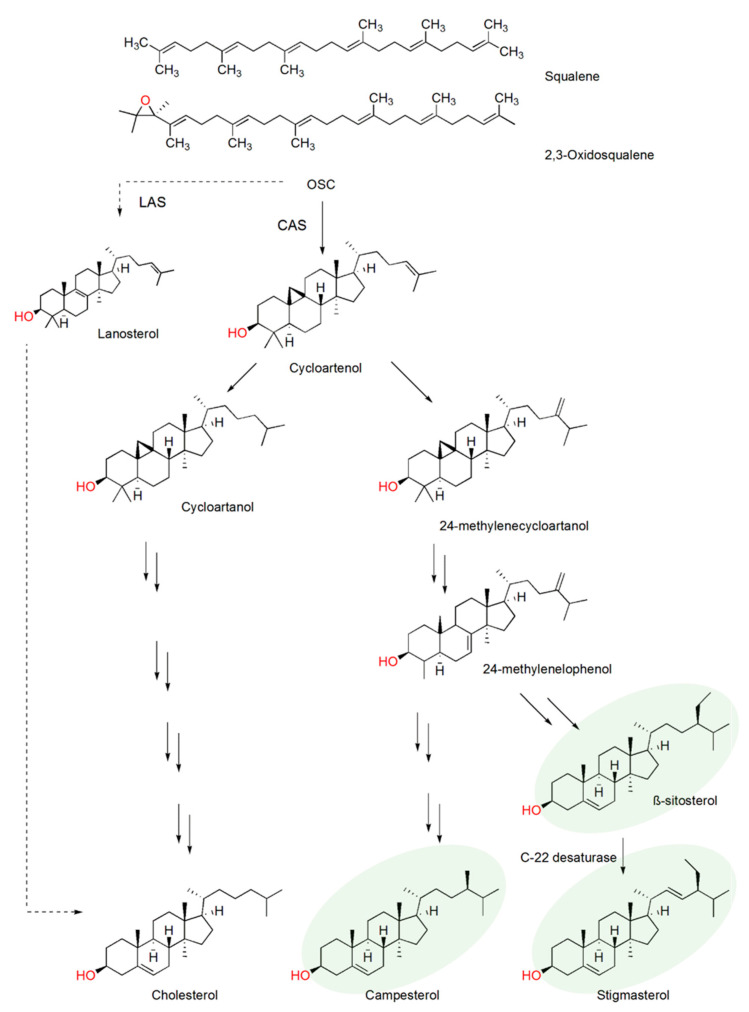
Plant sterol synthesis pathway starting with the conversion of 2,3 oxidosqualene to cycloartenol by oxidosqualene cyclase (OSC). OSC enzymes are classed as cycloartenol synthase (CAS) or lanosterol synthase (LAS) depending on their first cyclic product. The main sterol synthesis pathway in plants is indicated by multiple arrows representing several enzymatic steps with detailed information on β-sitosterol conversion to stigmasterol by a C22-desaturase. The most common end sterols in plants are highlighted in gray. The lanosterol synthesis pathway, well known for animal and fungi is indicated by dotted lines as lanosterol synthesis has been reported in plants, although lanosterol was not detected in this study.

Plants are consistently exposed to numerous pests and pathogens, which leads to variations in plant metabolism, including sterol profiles. Sterols are biomolecules which play important roles in various biological processes. Besides their essential function in cell membrane support and fluidity, they are also important as hormone precursors and are involved in biotic and abiotic stress responses [1,2,3,4,5]. Sterols belong to the large group of isoprenoid synthesized via the lanosterol (animals and fungi) or cycloartenol (plants) pathway (Figure 1), sharing a basic structure with a four-cyclic hydrocarbon ring, called gonane, and a hydroxyl group at position C-3. Depending on the organism, sterols are differently modified in the ring structure or in the side chain at position C-17, by methylations or double bonds [4,6]. Cholesterol, arguably the most studied sterol, is mainly synthesized in animals. In contrast, plants largely contain a mixture of C-24 sterols, such as β-sitosterol, campesterol and stigmasterol (collectively known as phytosterols). Nevertheless, they also synthesize minor amounts of cholesterol (Figure 1).

Remarkably not all multicellular organisms that require sterols for growth and reproduction are able to synthesize these molecules de novo [7]. Plant parasitic nematodes (PPN), for instance, are among the sterol auxotrophic parasites that rely on host plant sterols for growth and reproduction [7,8,9]. Several PPN are sedentary endoparasites that burrow inside plant roots and induce the formation of feeding sites, such as the root-knot nematodes, *Meloidogyne* spp. These nematodes induce the formation of giant cells in the differentiating vascular tissue that act as nutrient sinks, for example, sterols, which the nematode feeds on [10,11].

Biotic and abiotic factors have been reported to cause changes in plant sterol levels. Metabolic changes in β-sitosterol and stigmasterol levels have also been associated with fungal or bacterial infection and were related to the induction of signaling pathways leading to the synthesis of antimicrobial molecules and changes in membrane permeability [2,5,12,13,14,15]. Differences in the β-sitosterol/stigmasterol ratio have also been associated with resistance and susceptibility of tomato plants to *Meloidogyne incognita* [16]. Furthermore, studies of Hedin et al. [17] show changes in β-sitosterol/stigmasterol levels after *M. incognita* infection of cotton plant roots. Besides these biotic factors, abiotic stresses, such as drought and temperature, have also been reported to affect plant β-sitosterol and stigmasterol levels [5,18].

Stigmasterol is synthesized from β-sitosterol by a single desaturase reaction that occurs at position C22 of the sterol side chain, catalyzed by the enzyme sterol C22-desaturase that belongs to the cytochrome P450 710 family (EC 1.14.19.41; Figure 1) [19,20]. Little is known about the regulation of β-sitosterol and stigmasterol levels in roots during plant defense against PPN. Thus, understanding how plant sterols change after PPN infection and how these changes influence plant defense might help designing nematode-resistant or tolerant crops, possibly with an altered sterol profile. In this way, to better understand the role of plant sterol composition during nematode infection, we investigated the sterol composition of *Brassica juncea* (brown mustard), *Cucumis sativus* (cucumber)*, Glycine max* (soybean)*, Solanum lycopersicum* (tomato cv. Moneymaker and cv. Oskar) and *Zea mays* (corn), after infection with *M. incognita*. Furthermore, changes in sterol composition were tracked over time and expression levels of sterol C22-desaturase gene followed in tomato cv. Moneymaker.

## 2. Results and Discussion

### 2.1. Plant Sterol Composition

**Figure 2:**
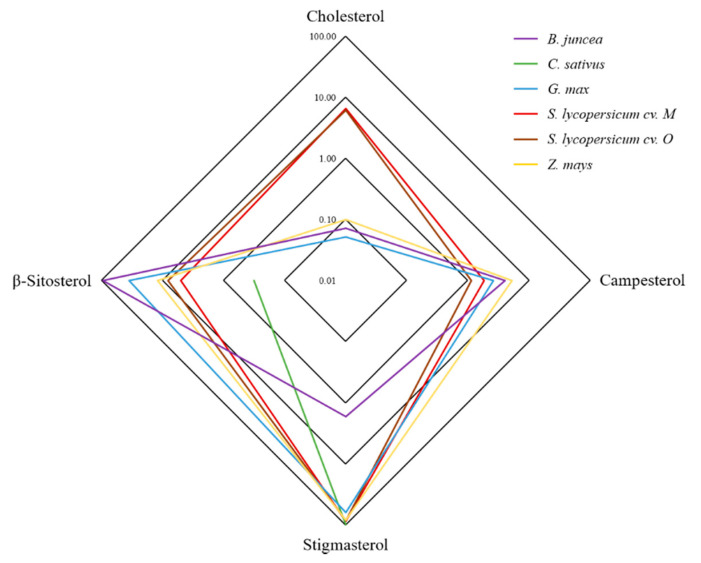
Free sterol composition in percentage of *Brassica juncea, Cucumis sativus, Glycine max, Solanum lycopersicum* cv. Moneymaker (M) and cv. Oskar (O) and *Zea mays*.

First, we investigated the profiles of free sterols in the roots of five different agricultural crops, brown mustard, corn, cucumber, soybean and two tomato cultivars (cv. Moneymaker and cv. Oskar) (Figure 2, Table 1). Notably, the cholesterol levels were significantly higher in both tomato cultivars than in the other four crop species. Brown mustard (*B. juncea*) had higher levels of β-sitosterol and lower levels of stigmasterol than all the other species. Significant sterol variations among vegetables, fruits, berries and medicinal plants have been reported [21,22,23]. However, data available for comparisons of plant root sterol composition are limited. With 80.7% stigmasterol in corn root systems, our data are in agreement with previous reports of Bladocha and Benveniste [24], which showed that sterol composition of corn roots and leaves differed strongly in the ratio of β-sitosterol to stigmasterol. Stigmasterol was the most abundant root sterol and β-sitosterol the most abundant sterol in leaves. In the medicinal plant Cannabis, significant differences in campesterol, β-sitosterol and stigmasterol have been observed between organs, with β-sitosterol as the most abundant sterol in stem bark and roots and stigmasterol being most abundant in leaves. Campesterol had the lowest concentration in roots and stem bark compared to β-sitosterol and stigmasterol [23].

Similar to our study where *B. juncea* sterols were composed of 94.1 % β-sitosterol (Figure 2; Table 1), the sterol composition from roots and leaves of the close relative *Brassica napus* is dominated by β-sitosterol [25]. On the other hand, Surjus and Durand [26] reported that β-sitosterol is the prominent plant sterol in roots of soybean cv. Hodgson, which does not match our findings where stigmasterol is the most abundant sterol with 62.4% in soybean cv. Aveline Bio.

*C. sativus* was the only species in this study where no campesterol was detected in the root sterol fraction, which was mainly composed of stigmasterol (Figure 2; Table 1). A study on the sterol composition of selected grains, legumes and seeds has shown that campesterol was also not detected in pumpkin seeds [27], whose sterols were mainly made up of β-sitosterol. In another study, neither campesterol, stigmasterol nor β-sitosterol were detected in *C. sativus* fruits, however other sterols were present [21]. Altogether, sterol compositions differ between organs of a plant, and even the same organs of different cultivars of the same species can differ significantly in their sterol composition and abundance [28].

Within plants, conjugated sterols are ubiquitous. However, their profile and relative content can differ among organs, plant developmental stage and environmental signals [29]. The analysis of total sterols (sterol ester and free sterols) and free sterol fraction is included in Table 1. When comparing the total sterol fraction to the free sterol fraction, the abundance of cholesterol and β-sitosterol increased, campesterol maintained a similar relative abundance, while the abundance of stigmasterol decreased. These results indicate that more cholesterol and β-sitosterol are present as steryl esters compared to stigmasterol. Overall, sterol profile changes have been reported for different tissues and conjugated forms [29] and even if a plant sterol, such as cholesterol represents a minor amount of the total sterol fraction of the plant, it can be the most abundant phytosterol in some tissue. For example, the sterol fraction of the phloem exudate of bean and tobacco plants contains over 88% of cholesterol [30].

### 2.2. Plant Sterol Composition after Meloidogyne Incognita Infection

**Figure 3:**
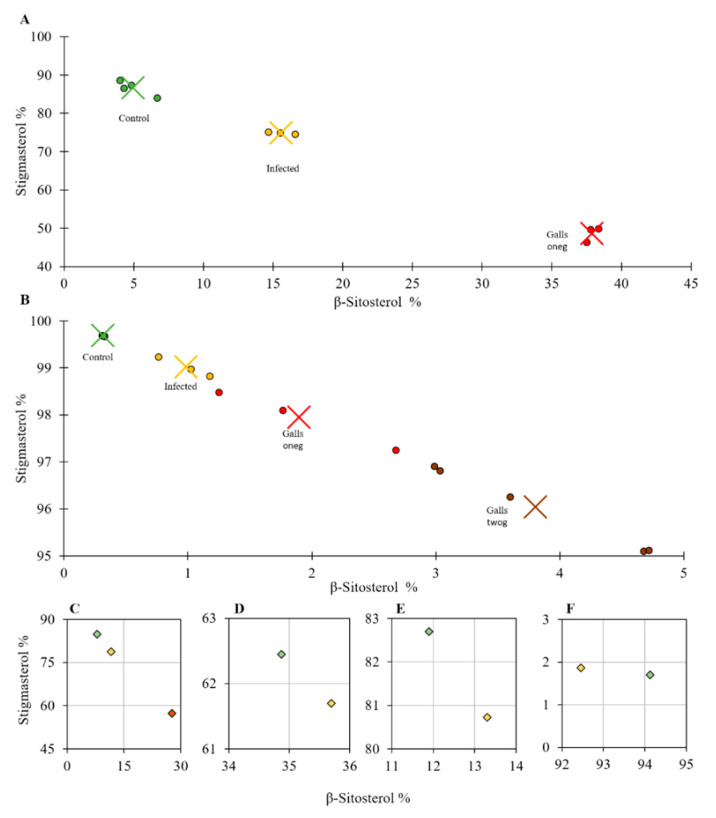
Relative stigmasterol to β-sitosterol abundance of uninfected (green), *M. incognita* infected (yellow) and galls (red) for one generation (oneg) and (brown) for second generation (twog) of *M. incognita*, samples of the plants: *Solanum lycopersicum* cv. Moneymaker (**A**) and *Cucumis sativus* (**B**). For *S. lycopersicum* cv. Oskar (**C**), *Glycine max* (**D**), *Zea mays* (**E**) and *Brassica juncea* (**F**) the results are presented as the mean of the 3 replicates. For A and B the average is marked by X. n = ≥3 replicates.

The sterol compositions of *M. incognita*-infected *B. juncea, C. sativus*, *G. max*, *S. lycopersicum* cvs. Oskar and Moneymaker, and *Z. mays* roots were determined 21 dpi (Table 1), to allow nematodes to establish and expand feeding sites [10]. Compared to uninfected tomato roots, sterols of cv. Moneymaker and cv. Oskar were composed of 6.5% and 6.1% free cholesterol, 86.7% and 84.7% stigmasterol, 5.0% and 8.0% β-sitosterol and 1.9% and 1.1% campesterol, respectively (Table 1). That means, infection with *M. incognita* led to an overall increase in cholesterol and β-sitosterol and a decrease in stigmasterol. Cholesterol levels increased up to 7.5% in cv. Moneymaker roots and up to 8.2% in cv. Oskar. The highest contribution of cholesterol to the sterol pool was determined in the galls, i.e., the nematode feeding sites, with 12.3% (cv. Oskar) and 10.3% (cv. Moneymaker; Table S2). Yet, the most pronounced sterol change observed 21 days post *M. incognita* inoculation was in the relative abundance of β-sitosterol and stigmasterol. In both tomato cultivars, levels of free β-sitosterol increased from 5.0% to 15.6% and 8.0% to 11.6% in cv. Moneymaker and cv. Oskar, respectively. At the same time, stigmasterol levels decreased from 86.7% to 75% and from 84.7% to 78.7% in infected roots of cv. Moneymaker and cv. Oskar, respectively. These changes in the β-sitosterol/stigmasterol ratio were even more pronounced when the sterol composition of the galls was evaluated (Figure 3; Table S2).

The analysis of the free and the total sterol fraction of *C. sativus*, *G. max* and *Z. mays* roots infected by *M. incognita* showed similar β-sitosterol, stigmasterol and cholesterol changes compared to the control plants (Table 1). Infection with *M. incognita* resulted in a relative increase in cholesterol and β-sitosterol combined with a relative decrease in stigmasterol levels compared to uninfected plants. However, such changes were not observed in *B. juncea*, where infection resulted in an increase in campesterol.

It seems plausible that the observed changes in the sterol pool are linked to a metabolic reaction against the infection by *M. incognita*. For example, in solanaceous plants, cholesterol can make up a significant portion of the overall sterol pool and has been suggested as a precursor of toxic steroidal alkaloids and glycoalkaloids [31]. Campesterol is used in numerous plants as precursor for the synthesis of brassinosteroid phytohormones, essential for the regulation of numerous plant processes, such as cell expansion and elongation, senescence and protection against drought and chilling [32]. The conversion of β-sitosterol to stigmasterol has been linked to biotic and abiotic stress [2,19,20] and has previously been linked to resistance against *M. incognita* in tomato cultivars [16].

As *M. incognita* induces a formation of giant cells, the galls sterol composition might be influenced by the lipid bilayer reorganization of these cells. Studies on the lipid bilayer revealed that β-sitosterol is slightly more efficient in ordering a fluid membrane of 2-dipalmitoyl-sn-glycero-3-phosphocholine than stigmasterol, resulting in a more packed membrane liquid ordered phase [33]. Furthermore, simulations have shown that cholesterol was slightly more efficient in packing the lipid bilayer than β-sitosterol [34].

Since the β-sitosterol to stigmasterol ratio is regulated by a single C22 desaturation step and strong changes in this ratio were observed, scatter plots were prepared to compare β-sitosterol/stigmasterol changes after nematode infection in the different plant species (Figure 3). All plant species analyzed displayed an increase of β-sitosterol and a decrease in stigmasterol after nematode infection, with the exception of *B. juncea*, which showed a decrease of β-sitosterol levels. β-Sitosterol accounted for 94.1% and stigmasterol for only 1.7% of free sterols in non-infected *B. juncea* plants (Table 1). This might be the reason why *B. juncea* displayed a completely different alteration on the sterol profile in response to nematode infection than the other plant species investigated (Table 1, Figure 2 and Figure 3). Anyhow, similar β-sitosterol/stigmasterol observations can be seen for other sterol analyses, e.g., of two cotton cultivars, cv. ST-213 and cv. 81-249 where the β-sitosterol/stigmasterol ratio changed from 32.6/53.1% (cv. St213) and 30.0/43.8% (cv. 81-249) to 36.8/43.8% (cv. ST-213) and 33.8/47.3% (cv. 81-249) after *M. incognita* infection [17].

A reason for the different sterol response in *B. juncea* compared to the other plant species might be that *Brassica* species contain a particular sterol, brassicasterol. Brassicasterol synthesis belongs to the same sterol branch as campesterol (Figure 1). The campesterol precursor 24-methyldesmosterol is converted to 24-epi-campesterol and then to brassicasterol. This final enzymatic step described in *Arabidopsis thaliana* is catalyzed by a C22 desaturase [19]. In this context, it is also important to note that *Brassica* species can produce isothiocyanates (ITCs) the glycosides of which are hydrolyzed by myrosinases in response to herbivory [35]. ITCs are highly toxic, leading to a suppressive effect of *Brassica* species on soil-borne pathogens and herbivores [36]. Therefore, *Brassica* species including *B. juncea* are used as cover crops in PPN management via so-called bio-fumigation [37,38]. Nevertheless, *B. juncea* is a host of *M. incognita* [39].

### 2.3. β-Sitosterol/Stigmasterol Conversion in Tomato after Meloidogyne Incognita Infection

**Figure 4:**
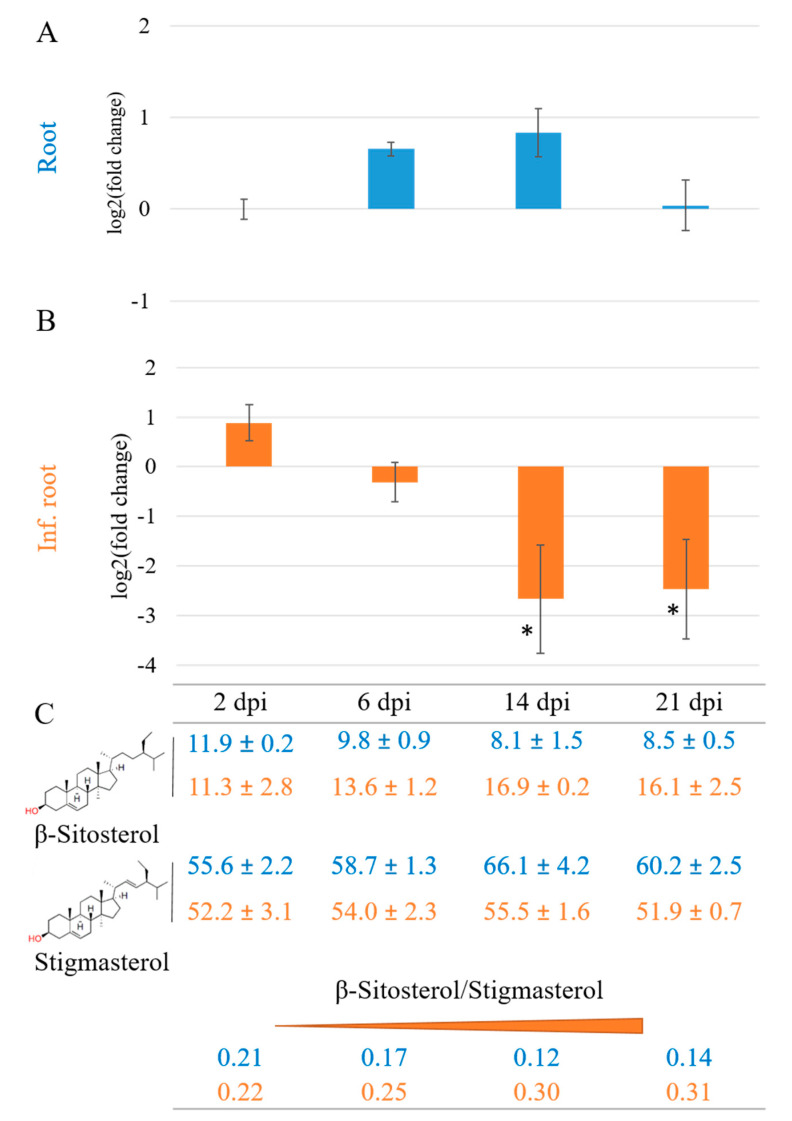
Temporal gene expression analysis of the C-22 desaturase gene *CYP710A11* and changes in the β-sitosterol/stigmasterol ratio in *Solanum lycopersicum* cv. Moneymaker 2, 6, 14 and 21 days post inoculation (dpi). *SlCYP710A11* gene expression is presented as fold change. (**A**) Data on uninfected roots are marked in blue. (**B**) Data on *M. incognita*-infected roots (Inf. roots) in orange. N = 4 biological replicates of 2 pooled plants per analysis. (**C**) Changes in β-sitosterol/stigmasterol ratios are displayed as percentage of total sterols extracted. ANOVA was used for comparisons of gene expression levels in uninfected vs. infected root systems. *, *p* < 0.05.

The β-sitosterol to stigmasterol conversion requires the creation of a double bond at position C22, which is catalyzed by a monooxygenase of the Cytochrome P450 enzyme family 710A (CYP710A), the only family in the CYP710 clan (Figure 1) [19,40]. The observed increase of β-sitosterol and decrease of stigmasterol led us to investigate the expression of the tomato gene *SlCYP710A11* during *M. incognita* infection. This gene encodes the enzyme previously characterized as a C22 desaturase in tomato sterol biosynthesis [19]. Temporal gene expression analysis of the *SlCYP710A11* gene in uninfected tomato cv. Moneymaker showed only small variations in gene expression levels during a time course of 21 days (Figure 4A). However, in tomato plants of the same developmental stage infected by *M. incognita*, the expression of *SlCYP710A11* was downregulated significantly in the samples taken at 14 and 21 dpi (Figure 4B). At the same time, the tomato sterol profile of β-sitosterol and stigmasterol reflected the gene expression levels (Figure 4C) in that the β-sitosterol/stigmasterol ratio gradually increased over the course of 21 days due to a relative increase of β-sitosterol and a corresponding decrease of stigmasterol (Figure 4C). The β-sitosterol/stigmasterol change was most pronounced at 21 dpi, confirming the previous results on plants infected with *M. incognita* that displayed reduced relative levels of stigmasterol and increased levels of β-sitosterol compared to the uninfected plants, most easily to explain by a decrease in C22 desaturase activity (Figure 4B). Interestingly, the change in the β-sitosterol/stigmasterol ratio was already visible at 6 dpi when transcriptional repression was not apparent yet, suggesting additional regulatory mechanisms (Figure 4B). Altogether, both gene expression and sterol profile data support the finding that the synthesis of stigmasterol from β-sitosterol is downregulated as an effect of *M. incognita* infection in *S. lycopersicum*.

Since the reaction to *M. incognita* infection is a modulation of C22 desaturase activity on behalf of the plants, it is important to note that the enzyme responsible for the conversion of 24-epi-campesterol to brassicasterol also represents a C22 desaturase; indeed, it was found for *Arabidopsis* that the enzyme encoded by *CYP710A2* was responsible for both brassicasterol and stigmasterol production [19]. However, *M. incognita* infection did not lead to a significant change in the sterol pattern of *B. juncea* (Table 1). Hence, in spite of the fact that brassicasterol was not analyzed, we can conclude that it is unlikely that the expression of the *CYP710A2* orthologue was affected.

Changes in the β-sitosterol/stigmasterol equilibrium might represent a general plant response to environmental cues as reviewed by Zhang et al. [28], and not a specific response to *M. incognita*. For example, an increase in stigmasterol levels has generally been observed as response to cold stress [5,41]. An increase in C22 desaturase expression levels has been reported as response of *Arabidopsis thaliana* plants to biotic and abiotic factors: to inducers of PAMP-triggered immunity like flagellin and lipopolysaccharides, to reactive oxygen species (ROS) and osmotic stress as well as to infections with bacterial and fungal pathogens [3,5,14,15,42]. Other than in *Arabidopsis*, a relative increase in stigmasterol has also been observed in leaves of *Triticum aestivum* infected by a biotrophic fungus, and in *Z. mays* leaves infected by a necrotrophic fungus ([43,44].

While our results seemed to show *CYP710A* gene induction at the first two time points of *M. incognita* infection, these changes were not significant. However, the repression of *SlCYP710A11* expression at 14 and 21 dpi, and the corresponding changes in the β-sitosterol/stigmasterol ratio, contrast with the abovementioned studies, where *CYP710A* expression was induced, β-sitosterol levels decreased and stigmasterol levels increased. It has to be kept in mind that most previous studies on plant sterol abundance during plant defense focused on shorter time intervals after exposure to pathogens, above-ground plant organs and were conducted mainly on *Arabidopsis* plants, where β-sitosterol is the most abundant sterol and brassicasterols make up part of the end sterols [14,15,43,44]. Furthermore, *Arabidopsis*, like *B. juncea*, is a member of the Brassicaceae and can produce nematocidal ITCs, which might affect its additional responses to PPN [45]. Altogether, the finding of an increase in the β-sitosterol/stigmasterol ratio in response to PPN infection in a diverse group of plants that do not produce nematocidal toxins might indeed represent a specific response. However, given that this response takes some time to establish, it is possible that it is not part of the defense against PPN, but of the supply of PPN with suitable sterols by the plant.

Given that plant-pathogen interactions are processes with different stages, in which gene expression levels often vary, it is not surprising to see changes in profiles of metabolites, such as sterols that could play a critical role in a plant-nematode interaction. It would also not be surprising that different pathogens/herbivores trigger similar or different plant responses. At this point, additional investigations have to be conducted to (a) compare the effects of PPN vs. other root pathogens/herbivores, and (b) evaluate the impact of the initial plant sterol composition on sterol changes after pathogen attack. After all, in the current study, *B. juncea* had the highest β-sitosterol abundance and was the only outlier in the sterol response to *M. incognita*, presumably due to the fact that Brassicaceae have particular sterol profiles including brassicasterol.

### 2.4. CYP710A

CYP710A represents the plant cytochrome P450 monooxygenase family encoding the sterol C22 desaturase, which is converting β-sitosterol to stigmasterol [40]. Like plants, fungi possess C22 desaturase enzymes known as CYP61 family of P450 enzymes, which are experimentally characterized and phylogenetically represent orthologues of the plant CYP710 protein family. Phylogenetic analysis of P450 diversity suggests that the CYP710 family is conserved from green algae to higher plants throughout evolution [46] and that the biochemical function can be traced back to plant-fungal divergence but was lost in animals [40]. During evolution, sterol 14-demethylase (CYP51) gene is assumed to have given rise to the *CYP710/CYP61* genes as their function in sterol biosynthesis is downstream of that of CYP51 [40]. CYP51 enzymes are present in plants, fungi and animals synthesizing sterols.

**Figure 5:**
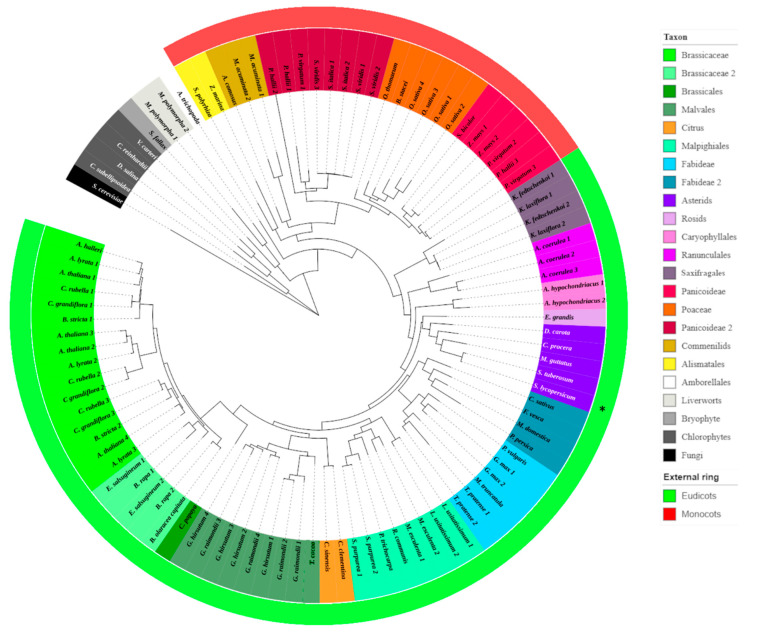
Phylogenetic maximum likelihood tree of the CYP710 enzyme family. The tree is rooted at ERG5, which is the *Saccharomyces cerevisiae* protein from which all CYP710 proteins originated [20]. Multiple sequences of the same plant species are numbered, and accession numbers of all selected proteins are reported in Table S2. Tree branches are colored and grouped by taxon. External ring shows the eudicots apart from the monocots. * Tomato CYP710A11 enzyme.

While the phylogeny of P450 monooxygenases is well researched, only limited phylogenetic information is available for CYP710 [28,40]. Overall, CYP710 enzyme activity and/or gene expression has only been studied in few plants, such as *A. thaliana* [2,14,19], *S. lycopersicum* [19], *Physcomitrella patens* [47] and *Calotropis procera* [48]. Therefore, we conducted a phylogenetic analysis of our studied tomato SlCYP710A11 protein and other plant CYP710 enzymes (Figure 5; Table S3). The well-studied AtCYP710A1 (*A. thaliana*) and SlCYP710A11 (*S. lycopersicum*) amino acid sequences were used as queries to mine for plant homologues. Four hits were scored in *A. thaliana*: Cytochrome P450 proteins 710A1, 710A2, 710A3 and 710A4 (NCBI accessions NP_180997.1, NP_180996.1, NP_180451.1 and NP_180452.1). It is worth mentioning that in *A. thaliana* both 710A1 and 710A2 can convert β-sitosterol to stigmasterol [19]. For *Z. mays*, two protein sequences were found in the NCBI database, from two different studies, one annotated as ‘uncharacterized protein’ and one as CYP710A11 (NP_001307723.1 and PWZ33314.1, respectively). For *G. max*, two proteins were identified, one annotated as CYP710A1 (XP_003542931.1) and one as CYP710A11 (XP_003546088.1). Only one homologous protein was found in *C. sativus* (XP_004134602.1), also annotated as CYP710A11 (Table S3). Since *B. juncea* sequences were not present in the NCBI or UniProt databases, *Brassica rapa* was used as a close relative.

During the blast search, multiple gene duplication events were observed, mostly at the species level (data not shown). The only duplication observed at the family level was found in the Brassicaceae family (whole genome duplication [49]). The phylogenetic analysis showed the divergence of eudicot and monocot CYP710 enzymes and basically followed plant phylogeny (Figure 5).

Based on the sterol analysis of the selected plants, the phylogenetic analysis, and recent studies (e.g., where *C. procera CYP710A* gene expression did not respond to abiotic factors [48]), we cannot conclude that in all plants C22 desaturase gene expression responds the same way to PPN infection. Moreover, not all CYP710A enzymes function the same way in sterol biosynthesis, and there might be undiscovered members of the CYP710A family catalyzing the same, or a different reaction (like the desaturation of 24-epi-campesterol to brassicasterol as reviewed by Zhang et al. [28]). Generally, among plant sterol synthesis enzymes, sterol methyl transferase (SMT), delta (24)-sterol reductase (DWF1) and CYP710A are assumed to adjust end sterol composition [28]. Altogether, further studies are required to address the questions if the observed β-sitosterol/stigmasterol changes are species-specific and how additional sterol related genes are involved in the activation of *CYP710A* and changes of the β-sitosterol/stigmasterol equilibrium, and to evaluate their impact on nematode performance. These data might help to develop new nematode-resistant cultivars able to maintain a sterol equilibrium that is not suitable for nematode development.

## 3. Materials and Methods

### 3.1. Nematode Inoculation and Plant Material

The root-knot nematodes, *Meloidogyne incognita* (isolate Reichenau 2, R2) were maintained at Agroscope (Wädenswil, Switzerland) on *S. lycopersicum* cv. Oskar. Greenhouse conditions were set at 22 ± 2 °C, 60% relative humidity (RH) and 16 h/8 h light/dark rhythm. Second-stage juveniles (J2) were extracted from heavily galled root systems using a mist chamber (PM 7/119). J2 were stored at 6 °C prior to use [50]. For sterol profiling a minimum of three biological replicates were used per treatment (negative and positive controls) and species:, *Brassica juncea* cv. Sareptasenf (P. H. Petersen), *Cucumis sativus* cv. Landgurken (Bigler Samen) *Glycine max* cv. Aveline Bio (UFA), *Solanum lycopersicum* cultivars (cvs.) Moneymaker (HILDA) and Oskar (Syngenta) and *Zea mays* cv. Grünschnittmais (UFA) were used. Seeds were pre-germinated (*B. juncea* 3–5 days, *C. sativus* 2–3 days, *G. max* 4–6 days, *S. lycopersicum* 4–6 days and *Z. mays* 5–6 days) in Petri dishes with 5 mm of tap water and then planted into 14 cm diameter plastic pots, using a 3:1 (vol/vol) silver sand:steamed soil mixture (sieved field soil from Cadenazzo, Switzerland). Greenhouse conditions were set to 22 ± 4 °C, 60% RH and 16 h:8 h light:dark rhythm. Three four-week-old plants of each species/cultivar were inoculated with 10,000 *M. incognita* (R2) J2 per pot.

### 3.2. Sterol Extraction and GC-MS Analysis

Infected and uninfected (control) plant roots were washed free of soil 21 days post inoculation (dpi). For “galls” sterol analysis, galled uproot systems were manually separated with a scalpel. Roots and galls were washed and the separated materials shock-frozen in liquid nitrogen, and ground to powder using mortar and pestle. Sterols were extracted according to Bligh and Dyer [51]. Each root-powder sample (1 g) was separated into two equal parts and total lipids were extracted in chloroform:methanol (2:1 *v*/*v*) for 1 h at 60 °C. One of the two lipid fractions was further saponified for extraction of free and esterified sterols. Saponification was performed as described by Dahlin et al. [52] (alkaline saponification with 2M KOH in 95% ethanol). Both lipid fractions (saponified and total lipid extract) of each root sample were dried under nitrogen and processed for sterol separation by suspending the dried samples in hexane and using a silica solid phase extraction (SPE) column (6 mL SiOH columns, Chromabond, Macherey Nagel, Düren, Germany) as described by Azadmard-Damirchi and Dutta [53]. Eluted sterols were dried under nitrogen and suspended in chloroform for sterol analysis on the Varian 450-GC coupled to a Varian 240-MS Ion Trap (GC-MS) (Darmstadt, Germany). The software VARIAN MS Workstation v. 6.9.3 was used for instrument control and data acquisition. A VARIANT FactorFour Capillary column VF-5 ms of 30 m length, 0.25 mm inner diameter, and 0.25 µm film thickness was used as stationary phase. Helium was used as carrier gas at a flow rate of 1.0 mL/min. Inlet temperature was set at 320 °C. 10 µL of the chloroform sample were injected. Initial GC temperature was set at 225 °C and ramped up to 300 °C at 1.5 °C/min. Temperature was maintained at 300 °C for 10 min before ramping to 320 °C with 5 °C/min, and finally remaining stable at 320 °C for 6 min. Transfer line was set to 270 °C and ion trap temperature was 150 °C. Ion trap was operated with electron ionization (EI) set at an ionization energy of 70 eV and scan mode selection (*m*/*z* 50–900) started after 5 min solvent delay. Sterol standards (cholesterol, campesterol, β-sitosterol and stigmasterol) were obtained from Sigma-Aldrich (St. Louis, MO, USA) and used to compare retention times, sterol fragmentation and for relative sterol quantification. The software R (v. 3.6.2; R core team, 2018) was used to perform Student’s *t*-tests (*t*-tests) and ANOVA (analysis of variance) tests on the data obtained to investigate the statistical differences between samples. *T*-tests were used when only infected and uninfected samples were compared, ANOVA was performed when gall samples were included in the comparison.

### 3.3. CYP710A11 Temporal Gene Expression Analysis

Tomato cv. Moneymaker plants were grown as described above. 4000 *M. incognita* J2/plant were inoculated by pipetting equal amounts of nematodes into four 5 cm deep holes next to three-week-old tomato plants. 8 Plants were used per time point and pooled in 4 groups of 2 plants each. Plant roots were harvested from infected and uninfected plants at 2, 6, 14 and 21 dpi, frozen in liquid nitrogen and stored at −20 °C before RNA extraction in liquid nitrogen using the Thermo Scientific GeneJET Plant RNA Purification Mini Kit (Waltham, MA, USA). Genomic DNA was removed from the isolated RNA using iScript DNase, followed by RNA quality testing by agarose gel electrophoresis and NanoDrop One One/OneC Microvolume UV-Vis Spectrophotometer measurements (Thermo Fisher Scientific, Reinach, Switzerland). cDNA synthesis was performed using the iScript cDNA synthesis kit (Bio-Rad, Hercules, CA, USA). The tomato gene coding sequence of *SlCYP710A11* was used to design qPCR primers with the online tool Primer3 (v. 4.1.0, Whitehead Institute for Biomedical Research), with the setting of 20 nt primer sequence length, 110 to 130 bases of amplified fragments, 50% GC content and 60 °C melting temperature. Primer sequences (Table S1) were BLASTed against WormBase and NCBI databases to check target specificity. The same parameters were used to design qPCR primers for the reference genes. NormFinder statistical algorithms were used to evaluate the housekeeping gene stability of actin, α-tubulin, SlCBL1, GADPH and eEF1-α. Primer efficiency was determined using the program Real-time PCR Miner [54]. qPCR analyses were carried out according to the 480 SYBR Green 1 Master mix (Roche, Basel, Switzerland) protocol and optimized to the primer melting temperature of 60 °C on the Roch LightCycler 480. For each qPCR run, the Roche LightCycler 480 program was used for melting peak and temperature evaluation. Each experiment was normalized according to the reference gene expression of actin and α-tubulin. Relative fold-changes in expression levels were analysed in Excel using 2^(−ΔΔCt)^ [55].

### 3.4. Phylogenetic Analysis of Cytochrome P450 Proteins

The protein sequences of *A. thaliana* AtCYP710A1 and *S. lycopersicum* SlCYP710A11, retrieved from the UniProtKB (UniProt) database, were used as queries in a sequence similarity search, performed on the UniProt and National Center for Biotechnology Information (NCBI) databases. The number of CYP710A1 proteins and their accession numbers were recorded for the plant species used in the sterol analysis. Protein sequences were searched for conserved protein domains using the Pfam (v. 32, European Bioinformatics Institute) and PANTHER protein databases. AtCYP710A1 was also used as query in a BLAST on Phytozome database (v12.1.5) [56]. Retrieved cytochrome P450 710 protein sequences were aligned using MUSCLE with the software MegaX (Molecular Evolutionary Genetics Analysis X). Aligned sequences were used in MegaX for phylogenetic analysis using the Maximum Likelihood approach, with 1000 bootstraps. The online tool iTOL (interactive Tree Of Life, v. 5.6) was used to finalize the phylogenetic tree.

## 4. Conclusions

In this study, we report changes in plant sterol profiles, in response to infection by the plant parasitic nematode *M. incognita*. The β-sitosterol/stigmasterol ratio in *C. sativus*, *G. max*, *S. lycopersicum* cv. Moneymaker and cv. Oskar and *Z. mays* were strongly affected by *M. incognita*. Interestingly, *B. juncea* revealed a sterol response different from that in the other plants examined. Since the conversion of β-sitosterol to stigmasterol is mediated by a single desaturation reaction at position C22 of the sterol side chain catalyzed by CYP710A, we investigated the transcriptional response of tomato *SlCYP710A11*. Infection of *S. lycopersicum* cv. Moneymaker with *M. incognita* led to repression of *SlCYP710A11* transcription that paralleled the change in the β-sitosterol/stigmasterol ratio. However, a detailed comparison indicates that the change in expression levels was not the only factor changing the sterol profile. Further studies are required to investigate whether the changes in plant sterol composition were specific to the response to *M. incognita* infection, if other nematode species generate the same changes in plant sterol composition, and whether they can represent a resistance mechanism.

## Figures and Tables

**Table 1:** Average percentage of free and total (in brackets) sterols of *Meloidogyne incognita* infected (Inf.) and non-infected brown mustard (*Brassica juncea*), cucumber (*Cucumis sativus*), soybean (*Glycine max*), tomato (*Solanum lycopersicum* cv. Moneymaker and cv. Oskar) and corn (*Zea mays*) roots.

Plant Species	Sample	Cholesterol	Campesterol	Stigmasterol	β-Sitosterol
***B. juncea***	Root	0.1 (0.1)	4.1 (5.6)	1.7 (1.6)	94.1 (92.7)
	Inf. root	0.1 (0.2)	5.6 (7.3)	1.9 (1.9)	92.5 (90.7)
	*p* value	0.79	0.07	0.72	0.12
***C. sativus***	Root	ND (0.1)	ND (ND)	99.7 (99.5)	0.3 (0.5)
	Inf. root	ND (0.2)	ND (ND)	99.0 (99.1)	1.0 (0.7)
	*p* value	NA	NA	0.03 *	0.03 *
***G. max***	Root	0.1 (0.3)	2.6 (2.3)	62.4 (56.5)	34.9 (40.9)
	Inf. root	0.1 (0.2)	2.6 (2.5)	61.7 (59.3)	35.7 (37.8)
	*p* value	0.95	0.07	0.45	0.41
***S. lycopersicum*** **cv. Moneymaker**	Root	6.5 (9.1)	1.9 (2.3)	86.7 (75.5)	5.0 (13.1)
	Inf. root	7.5 (11.4)	1.9 (3.0)	75.0 (65.6)	15.6 (20.0)
	*p* value	0.15	0.28	9.4 × 10^−4^ ***	5.1 × 10^−5^ ***
***S. lycopersicum*** **cv. Os** **kar**	Root	6.1 (7.3)	1.1 (1.5)	84.7 (80.3)	8.0 (10.9)
	Inf. root	8.2 (9.8)	1.5 (1.7)	78.7 (75.4)	11.6 (13.2)
	*p* value	0.1	0.02 *	0.07	0.09
***Z. mays***	Root	0.1 (0.2)	5.3 (5.3)	82.7 (81.2)	11.9 (13.3)
	Inf. root	0.2 (0.3)	5.8 (5.5)	80.7 (80.7)	13.3 (13.5)
	*p* value	0.05 *	0.09	0.1	0.003 **

Student’s *t*-test was used for comparisons of uninfected vs. infected root systems. ***, *p* < 0.001; **, *p* < 0.01; *, *p* < 0.05. ND = not detected. n = minimum of 3 samples.

## Data Availability

Data is contained within the article or supplementary material.

## Supplementary Materials

The following are available online at https://www.mdpi.com/2223-7747/10/2/292/s1, Table S1: Primer pairs used for qPCR analysis of tomato (*Solanum lycopersicum*), Table S2: Sterol composition (%) of tomato (*Solanum lycopersicum*) and cucumber (*Cucumis sativus*) galls caused by *Meloidogyne incognita*, Table S3: List of CYP710 enzyme sequences used for the phylogenetic analysis.
