# Supplementary material for: Changes in the Plant β-Sitosterol/Stigmasterol Ratio Caused by the Plant Parasitic Nematode Meloidogyne incognita
Source: Plants (Basel). 2021 Feb 4;10(2):292. doi: 10.3390/plants10020292 (PMC7913658; doi:10.3390/plants10020292)
Supplement: Supplementary file 1 [file plants-10-00292-s001.pdf]

Supplementary Material

1 Supplementary Tables

Supplementary Table S1. Primer pairs used for qPCR analysis of tomato (*Solanum lycopersicum*).

GENE	ACCESSION NUMBER	FORWARD PRIMER	REVERSE PRIMER
CYP710A11	NM_001247585.2	TCGACTTCCTCTTTGCTGCT	CGCTACATCCGACCGAACTT
A-TUBULIN	NM_001347592.1	ATGTTCCCCGTGCTGTCTTT	CTTTGCCACTGATGAGCTGC
ACTIN	NM_001308447.1	AAGTGCGAGTGTCCTGTCTG	TACCGTGCATTCATAGCCCC
SLCBL1	NM_001252118.1	TTTGGTGATTTCGTGAAGGGAC	TCTCAATGAACCCTGTGCCA
GAPDH	NM_001247874.2	ACCACAAATTGCCTTGCTCC	GGTCTTCTGAGTGGCTGTGA
EEF1-A	NM_001247106.2	GATGATTCCCACCAAGCCCA	TCTTGACAACACCGACAGCA

Supplementary Table S2.
Sterol composition (%) of tomato (*Solanum lycopersicum*) and cucumber (*Cucumis sativus*) root knot galls caused by *Meloidogyne incognita*.

SPECIES	CHOLESTEROL %	CAMPESTEROL %	STIGMASTEROL %	β-SITOSTEROL %
S. lycopersicum cv. M	10.34	3.00	48.79	37.87
S. lycopersicum cv.O	12.26	2.75	57.22	27.77
C. sativus oneg	0.16	ND	97.95	1.90
C. sativus twog	0.16	ND	96.04	3.80

Solanum lycopersicum cv. Moneymaker (M), *Solanum lycopersicum cv. Oskar* (O) *Cucumis sativus* root knot galls from one generation (oneg) and two generations (twog). Percentage are means of minimum three biological replicates. ND = not detected.

Supplementary Table S3. List of CYP710 enzyme sequences used for the phylogenetic analysis.
"Number" represents the numbering of the phylogenetic tree in the main manuscript.

Taxon	Species	Number	Phytozome accession code
Ranunculales	*A. coerulea*	1	Aqcoe4G009200.1.p
Ranunculales	*A. coerulea*	2	Aqcoe4G236600.1.p
Ranunculales	*A. coerulea*	3	Aqcoe4G299500.1.p
Commelinids	*A. comosus*		Aco010658.1
Brassicaceae	*A. halleri*		Araha.16206S0001
Caryophyllales	*A. hypochondriacus*	1	AHYPO.007104
Caryophyllales	*A. hypochondriacus*	2	AHYPO.008253
Brassicaceae	*A. lyrata*	1	AL4G30460.t1
Brassicaceae	*A. lyrata*	2	AL4G23390.t1
Brassicaceae	*A. lyrata*	3	AL4G30470.t1

Brassicaceae	*A. thaliana*	1	AT2G34490.1
Brassicaceae	*A. thaliana*	2	AT2G28850.1
Brassicaceae	*A. thaliana*	3	AT2G28860.1
Brassicaceae	*A. thaliana*	4	AT2G34500.1
Amborellales	*A. trichopoda*		evm.TU.AmTr v1.0 scaffold00141.11
Brassicaceae 2	*B. olaracea capitata*		Bol027531
Brassicaceae 2	*B. rapa*	1	Brara.D02097
Brassicaceae 2	*B. rapa*	2	Brara.E00976.1.p
Grass	*B. stacei*		Brast01G342000.1.p
Brassicaceae 2	*B. stricta*	1	Bostr.23794s0661
Brassicaceae 2	*B. stricta*	2	Bostr.23794s0659.1.p
Citrus	*C. clementina*		Ciclev10014973m
Brassicaceae 2	*C. grandiflora*	1	Cagra.7352s0002
Brassicaceae 2	*C. grandiflora*	2	Cagra.1960s0002
Brassicaceae 2	*C. grandiflora*	3	Cagra.7352s0001.1.p
Brassicales	*C. papaya*		evm.TU.supercontig_166.19
Asterids	*C. procera*		AYA73282.1
Chlorophyte	*C. reinhardtii*		Cre11.g467527.t1.1
Asterids	*C. rubella*	1	Carubv10024564m.g
Asterids	*C. rubella*	2	Carubv10024647m.g
Asterids	*C. rubella*	3	Carubv10024737m
Fabidae	*C. sativus*		Cucsa.047670.1
Citrus	*C. sinensis*		orange1.1g046882m
Chlorophyte	*C. subellipsoidea*		estExt
Asterids	*D. carota*		DCAR_027922
Chlorophyte	*D. salina*		Dusal.0187s00006.1.p
Rosid	*E. grandis*		Eucgr.D00302.1.p
Citrus	*E. salsugineum*	1	Thhalv10016552m
Citrus	*E. salsugineum*	2	Thhalv10016547m
Fabidae	*F. vesca*		gene30586-v1.0-hybrid
Malvales	*G. hirsutum*	2	XP_016688145.1
Malvales	*G. hirsutum*	1	XP_016683544.1
Malvales	*G. hirsutum*	3	XP_016731828.1
Malvales	*G. hirsutum*	4	XP_016688146.1
Fabidae 2	*G. max*	1	Glyma.13G217400.1.p
Fabidae 2	*G. max*	2	Glyma.15G095000.1.p
Malvales	*G. raimondii*	1	Gorai.010G166000.1
Malvales	*G. raimondii*	2	Gorai.010G166100.1
Malvales	*G. raimondii*	3	Gorai.009G155600.1
Malvales	*G. raimondii*	4	Gorai.009G155800.1
Crassulaceae	*K. fedtschenkoi*	1	Kaladp0395s0043.1.p
Crassulaceae	*K. fedtschenkoi*	2	Kaladp0840s0006.1.p
Crassulaceae	*K. laxiflora*	1	Kalax.0164s0003.1.p
Crassulaceae	*K. laxiflora*	2	Kalax.0554s0026.1.p
Malpighiales	*L. usitatissimum*	1	Lus10028129
Malpighiales	*L. usitatissimum*	2	Lus10042833.g

Commelinids	*M. acuminata*	2	Achr10G14330.001
Commelinids	*M. acuminata*	1	Achr8G20010.001
#BEZUG!	*M. domestica*		MDP0000945293
Malpighiales 2	*M. esculenta*	2	Manes.01G172800.1.p
Malpighiales 2	*M. esculenta*	1	Manes.02G132100.1.p
Asterids	*M. guttatus*		Migut.H01366.1.p
Liverworts	*M. polymorpha*	1	Mapoly0045s0151.1.p
Liverworts	*M. polymorpha*	2	Mapoly0103s0038.1.p
Fabidae 2	*M. truncatula*		Medtr2g019640.1
Poaceae	*O. sativa*	1	LOC_Os01g11270
Poaceae	*O. sativa*	2	LOC_Os01g11280
Poaceae	*O. sativa*	3	LOC_Os01g11300
Poaceae	*O. sativa*	4	LOC_Os01g11340
Poaceae	*O. thomaeum*		Oropetium_20150105_17908
Panicoideae	*P. hallii*	1	Pahal.B00576.1
Panicoideae	*P. hallii*	2	Pahal.C00816.1
Panicoideae	*P. hallii*	3	Pahal.E04424.1
Fabidae	*P. persica*		Prupe.4G079400.1.p
Malpighiales	*P. trichocarpa*		Potri.004G131700.1
Panicoideae	*P. virgatum*	1	Pavir.la03059.1.p
Panicoideae	*P. virgatum*	2	Pavir.J35582.1.p
Panicoideae	*P. virgatum*	3	Pavir.Ea00174
Fabidae 2	*P. vulgaris*		Phvul.006G163800.1.p
Malpighiales 2	*R. communis*		28223.m000100
Panicoideae 2	*S. bicolor*		Sobic.003G021900.1.p
Fungi	*S. cerevisiae*		ERG 5 P54781
Bryophyte	*S. fallax*		Sphfalx0121s0046.1.p
Panicoideae 2	*S. italica*	1	Seita.9G366300.1.p
Panicoideae 2	*S. italica*	2	Seita.9G366300.2.p
Asterids	*S. lycopersicum*		Solyc02g070580.1.1
Lycophytes	*S. moellendorffii*		75218
Alismatales	*S. polyrhiza*		Spipo8G0071000
Malpighiales	*S. purpurea*	2	SapurV1A.0560s0090.1.p
Malpighiales	*S. purpurea*	1	SapurV1A.1504s0030.1.p
Asterids	*S. tuberosum*		PGSC0003DMG400021157
Panicoideae	*S. viridis*	2	Sevir.9G372000.2.p
Panicoideae	*S. viridis*	1	Sevir.9G372000
Panicoideae	*S. viridis*	3	Sevir.9G372000.3.p
Malvales	*T. cacao*		Thecc1EG029501t1
Fabidae 2	*T. pratense*	1	Tp57577_TGAC.v2.gene31185
Fabidae 2	*T. pratense*	2	Tp57577_TGAC.v2.gene35511
Chlorophyte	*V. carteri*		Vocar.0014s0235.1.p
Alismatales	*Z. marina*		Zosma88g00340.1
Panicoideae 2	*Z. mays*	1	AC233853.1.FGP003
Panicoideae 2	*Z. mays*	2	Zm00008a010737 ZmaysPH207
